# Endogenous erythropoietin at birth is associated with neurodevelopmental morbidity in early childhood

**DOI:** 10.1038/s41390-021-01679-0

**Published:** 2021-08-31

**Authors:** Elina J. Rancken, Marjo P. H. Metsäranta, Mika Gissler, Leena K. Rahkonen, Leena M. Haataja

**Affiliations:** 1 Children’s Hospital, Pediatric Research Centre, Helsinki University Hospital, University of Helsinki, Helsinki, Finland; 2 Information Services Department, Finnish Institute for Health and Welfare, Helsinki, Finland; 3 Department of Neurobiology, Care Sciences and Society, Karolinska Institute, Stockholm, Sweden; 4 Department of Obstetrics and Gynecology, Helsinki University Hospital, University of Helsinki, Helsinki, Finland

## Abstract

**Background:**

New biomarkers that predict later neurodevelopmental morbidity are needed. This study evaluated the associations between umbilical cord serum erythropoietin (us-EPO) and neurodevelopmental morbidity by the age of 2–6.5 years in a Finnish cohort.

**Methods:**

This study included 878 non-anomalous children born alive in 2012 to 2016 in Helsinki University Hospitals and whose us-EPO concentration was determined at birth. Data of these children were linked to data from the Finnish Medical Birth Register and the Finnish Hospital Discharge Register. Neurodevelopmental morbidity included cerebral palsy, epilepsy, intellectual disability, autism spectrum disorder, sensorineural defects, and minor neurodevelopmental disorders.

**Results:**

In the cohort including both term and preterm children, us-EPO levels correlated with gestational age (*r* = 0.526) and were lower in premature children. High us-EPO levels (>100 IU/l) were associated with an increased risk of severe neurodevelopmental morbidity (OR: 4.87; 95% CI: 1.05–22.58) when adjusted for the gestational age. The distribution of us-EPO levels did not differ in children with or without the later neurodevelopmental diagnosis.

**Conclusions:**

Although high us-EPO concentration at birth was associated with an increased risk of neurodevelopmental morbidity in early childhood, the role of us-EPO determination in clinical use appears to be minor.

**Impact:**

- We determined whether endogenous umbilical cord serum erythropoietin would be a new useful biomarker to predict the risk of neurodevelopmental morbidity.
- This study evaluated the role of endogenous erythropoietin at birth in neurodevelopmental morbidity with a study population of good size and specific diagnoses based on data from high-quality registers.
- Although high umbilical cord serum erythropoietin concentration at birth was associated with an increased risk of neurodevelopmental morbidity in early childhood, the clinical value of erythropoietin determination appears to be minor.

## Introduction

Human erythropoietin (EPO), a hormone whose synthesis increases under hypoxic conditions, regulates red blood cell production from the fetal stage.^1^ Elevated EPO levels in fetal plasma correlate well with the intensity of fetal hypoxia,^2^ as EPO does not pass through the placenta^3^ and is not stored in tissues.

According to preclinical studies, EPO has many neuroprotective effects.^4^ Preliminary results using EPO as a neuroprotective agent were promising in neonatal hypoxic–ischemic encephalopathy,^5^ and showed an improvement in cognitive outcome in very preterm infants.^6,7^ However, two randomized, placebo-controlled trials from 2016 and 2020 reported that, in extremely and very preterm infants, high-dose EPO treatment did not decrease the risk of neurological impairment at 2 years of age assessed using the Bayley Scales of Infant Development.^8,9^ In a further study in very preterm infants, the neurodevelopmental outcome showed no difference later at 5 years of age either.^10^

As for endogenous EPO, high endogenous EPO concentration in blood during the first postnatal days has been associated with neonatal morbidity in extremely preterm infants^11^ and with neonatal encephalopathy, abnormal MRI and mortality in term infants exposed to perinatal asphyxia.^12^ Minimal data exist on the association between endogenous EPO levels at birth and long-term neurodevelopmental morbidity in children. One follow-up study from 1988 reported that high fetal plasma EPO levels were associated with an increased risk of cerebral palsy or death at 2 years of age.^13^ Further, Korzeniewski et al. found that high EPO levels on a postnatal day 14 are associated with adverse outcomes (very low mental and psychomotor development indices and microcephaly) at 2 years of age in extremely preterm infants.^14^

Given the role of EPO in hypoxic–ischemic insult, we evaluated whether elevated umbilical cord serum EPO (us-EPO) levels at birth are associated with increased risks of severe neurodevelopmental morbidity by the age of 2–6.5 years in a Finnish cohort including both term and preterm children. Major neurodevelopmental morbidity included cerebral palsy, epilepsy, intellectual disability, autism spectrum disorder, and sensorineural defects consisting of visual and hearing impairments. The secondary aim was to assess the association of us-EPO with minor neurodevelopmental disorders in early childhood and infant mortality by the age of 1 year.

## Patients and methods

### Population and data sources

This register-based retrospective study included children born between 1 May 2012 and 31 December 2016 in maternity hospitals in Helsinki University Hospital and whose us-EPO concentration was determined from umbilical cord blood at birth (*n* = 959). Umbilical blood samples are routinely collected from every newborn immediately after birth. During the study period, us-EPO levels were determined systematically as a standard of care of newborns with suspected asphyxia (*n* = 331) based on a 1-min Apgar score of <4. In addition, with the separate consent of the parent, a determination was made from samples of newborns with Apgar score >3.

The baseline characteristics of these children were collected from the Finnish Medical Birth Register (MBR).^15^ We excluded 2 (0.2%) stillbirths and 79 (8.2%) children with major congenital anomaly defined in the Register on Congenital Malformations. Data of the 878 children that met the inclusion criteria were linked to the Finnish Hospital Discharge Register (HDR)^16^ and the Register on Visual Impairments.^17^

All registers are maintained by the Finnish Institute for Health and Welfare (THL). The MBR contains information on the mother’s health and interventions during pregnancy and delivery and the newborn infant’s care and outcome during the first week of life. This register contains data on all live births and stillbirths from gestational age of 22 + 0 weeks onwards or with a birth weight of at least 500 g. Data provided by the birth hospitals are completed by basic information from the Central Population Register and the Cause of Death Register maintained by Statistics Finland. The HDR records information on admission and discharge dates, main and secondary diagnoses, and surgical procedures for all inpatient care and public hospital outpatient care, covering over 95% of discharges in Finland.^16^ We used these data to complete information on the mothers’ pregnancy-related and delivery-related diagnoses and children’s diagnoses. Diagnoses of visual impairments were confirmed from the Register on Visual Impairments, which maintains a record of the prevalence and care of visual impairments.

### Classification

The study population was divided into subgroups according to the us-EPO levels. The us-EPO categories were low (us-EPO < 20 IU/l, *n* = 218), average (us-EPO 20–39 IU/l, *n* = 218), elevated (us-EPO 40–100 IU/l, *n* *=* 215), and high (us-EPO > 100 IU/l, *n* *=* 227). We chose us-EPO 20–39 IU/l as the reference group based on studies showing the mean or median fetal EPO level to be within this range at the end of uncomplicated term pregnancies.^18–21^

Apgar scores were provided by midwives or pediatricians according to standardized procedures. The 1- and 5-min Apgar scores were classified into the following three groups: low (0–3), intermediate (4–6), and normal (7–10).^22^ In 875 (99.7%) births, data were available for the 1-min score. For 236 (26.9%) births, data were missing for the 5-min Apgar score. A common practice in maternal hospitals of the Helsinki University Hospital is that the 5-min score is not recorded if the 1-min score is high (>7) and the child remains in good condition. If the 1-min Apgar score was normal (7–10), the 5-min Apgar score was substituted by the score at 1 min. Newborns were divided into the following subgroups by gestational weeks: early preterm (≤31 + 6) late preterm (32 + 0–36 + 6), term (37 + 0–41 + 6), and post-term (≥ 42 + 0). Gestational age was determined by the first-trimester ultrasound screening, as is routine practice in Finland.

### Outcome diagnoses

Children were followed until the end of 2018, when they were 2–6.5 years old. Neurodevelopmental impairment was recorded if at the end of 2018 the child was recorded in the HDR with *the International Statistical Classification of Diseases and Related Health Problems, 10th Revision* (ICD-10) codes for one or more of the following major or minor diagnoses. Major morbidity included cerebral palsy (G80.0–G80.9), epilepsy (G40.0–G40.9), intellectual disability (F70.0–F73.9 and F78.0–F79.9), autism spectrum disorder (F84.0–F84.9), and sensorineural defects consisting of visual impairment (H53.0–H53.4 and H54.0–H54.7) and hearing impairment (H90.0–H90.8). Minor morbidity included developmental disorders consisting of specific developmental disorders of speech and language (F80.1–80.9), a specific developmental disorder of motor function (F82), mixed specific developmental disorder (F83), and hyperkinetic disorders (F90.0–F90.9). Children with delayed development without a more specific diagnosis yet (R62.0) were analyzed separately. Data on birth asphyxia (P21.0–P21.9) are presented in Table 1.

**Table 1:** Characteristics of the study population, overall and stratified by us-EPO categories.

	Total	us-EPO level at birth						
		Low < 20		Average 20–39	Elevated 40–100		High > 100	
Characteristics	*n* (%)	*n* (%)	*p*-value^a^	*n* (%) Reference group	*n* (%)	*p*-value^a^	*n* (%)	*p*-value^a^
Total	878 (100.0)	218 (100.0)		218 (100.0)	215 (100.0)		227 (100.0)	
Mode of delivery								
Vaginal delivery	329 (37.5)	107 (49.1)	0.113	104 (47.7)	78 (36.3)	0.103	40 (17.6)	**<0.001**
Breech vaginal delivery	20 (2.3)	4 (1.8)		5 (2.3)	7 (3.3)		4 (1.8)	
Vacuum extraction/forceps	193 (22.0)	45 (20.6)		53 (24.3)	58 (27.0)		37 (16.3)	
Planned cesarean section	9 (1.0)	7 (3.2)		0 (0.0)	1 (0.5)		1 (0.4)	
Acute cesarean section	220 (25.1)	29 (13.3)		35 (16.1)	53 (24.7)		103 (45.4)	
Emergency cesarean section	107 (12.2)	26 (11.9)		21 (9.6)	18 (8.4)		42 (18.5)	
Male	498 (56.7)	127 (58.3)	0.178	113 (51.8)	127 (59.1)	0.130	131 (57.7)	0.213
Multiple birth	33 (3.8)	14 (6.4)	0.189	8 (3.7)	5 (2.3)	0.413	6 (2.6)	0.535
Gestational age (wk)								
<32	23 (2.6)	14 (6.4)	**<0.001**	3 (1.4)	3 (1.4)	0.117	3 (1.3)	**0.004**
32 + 0–36 + 6	77 (8.8)	28 (12.8)		13 (6.0)	7 (3.3)		29 (12.8)	
37 + 0–41 + 6	676 (77.0)	169 (77.5)		179 (82.1)	167 (77.7)		161 (70.9)	
≥42 + 0	102 (11.6)	7 (3.2)		23 (10.6)	38 (17.7)		34 (15.0)	
Birth weight (g, mean ± SD)	3387 (701)	3224 (814)	**<0.001**	3504 (610)	3517 (665)	0.831	3307 (658)	**0.001**
<2500	73 (8.3)	25 (11.5)	**0.025**	12 (5.5)	16 (7.4)	0.412	20 (8.8)	0.177
≥4500	20 (2.3)	3 (1.4)	0.475	5 (2.3)	6 (2.8)	0.742	6 (2.6)	0.812
Birth weight (g)								
SGA	60 (6.8)	10 (4.6)	0.401	14 (6.4)	10 (4.7)	0.421	26 (11.5)	0.064
LGA	26 (3.0)	10 (4.6)	0.630	8 (3.7)	5 (2.3)	0.413	5 (2.2)	0.358
1-min Apgar score								
0–3	331 (37.7)	61 (28.0)	0.690	71 (32.6)	94 (43.7)	0.068	105 (46.3)	**0.001**
4–6	193 (22.0)	41 (18.8)		43 (19.7)	41 (19.1)		68 (30.0)	
7–10	351 (40.0)	115 (52.8)		103 (47.2)	80 (37.2)		53 (23.3)	
No information	3 (0.3)	1 (0.5)		1 (0.5)	0 (0.0)		1 (0.4)	
5-min Apgar score								
0–3	71 (8.1)	16 (7.3)	0.998	15 (6.9)	16 (7.4)	0.450	24 (10.6)	**0.001**
4–6	214 (24.4)	40 (18.3)		41 (18.8)	51 (23.7)		82 (36.1)	
7–10	590 (67.2)	161 (73.9)		161 (73.9)	148 (68.8)		120 (52.9)	
No information	3 (0.3)	1 (0.5)		1 (0.5)	0 (0.0)		1 (0.4)	
Umbilical artery pH								
<7.00	75 (8.5)	12 (5.5)	0.314	19 (8.7)	24 (11.2)	0.426	20 (8.8)	0.570
7.00–7.10	144 (16.4)	28 (12.8)		34 (15.6)	41 (19.1)		41 (18.1)	
≥7.11	609 (69.4)	165 (75.7)		157 (72.0)	139 (64.7)		148 (65.2)	
No information	50 (5.7)	13 (6.0)		8 (3.7)	11 (5.1)		18 (7.9)	
Birth asphyxia	191 (21.8)	32 (14.7)	0.103	45 (20.6)	50 (23.3)	0.511	64 (28.2)	0.064
Admission to neonatal unit	349 (39.7)	69 (31.7)	0.678	65 (29.8)	75 (34.9)	0.260	140 (61.7)	**<0.001**

Data are presented as variables and percentage or mean ± standard deviation.

Bold values indicate statistical significance *p* < 0.05.

*SD* standard deviation, *SGA* small for gestational age, *LGA* large for gestational age, *us-EPO* umbilical cord serum erythropoietin.

^a^Test for relative proportions, Chi-square test or *t*-test. Reference group is us-EPO 20–39.

In Finland, all preschool children are invited, and practically all children (99.6%) attend, to an annual medical and developmental check-up free of charge.^23^ If in these check-ups child’s development raises concerns, the child will be referred to the pediatric neurology units of either secondary or tertiary care public hospitals for a more detailed assessment. A specialist sets diagnoses based on medical history, clinical multidisciplinary evaluation, brain imaging, and clinical neurophysiological investigations when appropriate. The most severe neurodevelopmental disorders (cerebral palsy, severe intellectual disability, deafness, and blindness) usually occur and are accordingly diagnosed within the first 2 years of life. Epilepsy is diagnosed at the time of the first symptoms, and intellectual disability and autism spectrum disorder are mostly diagnosed at the latest by the beginning of school at 6 years of age. The diagnosis “delayed milestone in childhood” is set when the child’s development is not age-appropriate, but a more accurate diagnosis cannot yet be made. Diagnoses are included in the HDR as soon as they are established. Data on deaths were available in the MBR only for the first year of life.

### Ethics

For this study, the THL, the hospital district of Helsinki and Uusimaa, and the Ethics Committee for gynecology and obstetrics, pediatrics, and psychiatry of Helsinki University Hospital provided the authorizations (THL/317/5.05.00/2019; §12/28.2.2018/HUS/185/2018; §181/24.8.2017/HUS/2313/2017) required by the data protection legislation in Finland. All register and hospital data linkages were performed using unique personal identification numbers anonymized by the authorities.

### Statistical analysis

Characteristics of newborns and their mothers are shown as a number of values with percentages for categorical variables and as means with standard deviations for normally distributed continuous variables. us-EPO level categories were compared with each other by test for relative proportions, chi-square test, or *t*-test when appropriate (Tables 1 and 2).

**Table 2:** Adverse outcome according to us-EPO categories.

		us-EPO level at birth						
	Total	Low < 20		Average 20–39	Elevated 40–100		High > 100	
Outcome	*n* (%)	*n* (%)	*p*-value^*a*^	*n* (%) Reference group	*n* (%)	*p*-value^a^	*n* (%)	*p*-value^a^
Total	878 (100.0)	218 (100.0)		218 (100.0)	215 (100.0)		227 (100.0)	
Infant mortality								
Died at the age of 0–364 days	12 (1.4)	3 (1.4)	0.653	2 (0.9)	3 (1.4)	0.642	4 (1.8)	0.440
*Morbidity, major*								
Any major impairment	26 (3.0)	9 (4.1)	**0.033**	2 (0.9)	5 (2.3)	0.245	10 (4.4)	**0.023**
Cerebral palsy	6 (0.7)	2 (0.9)	0.562	1 (0.5)	0 (0.0)	0.320	3 (1.3)	0.335
Epilepsia	5 (0.6)	1 (0.5)	1.000	1 (0.5)	2 (0.9)	0.554	1 (0.4)	0.977
Intellectual disability	7 (0.8)	2 (0.9)	0.156	0 (0.0)	3 (1.4)	0.080	2 (0.9)	0.165
Autism spectrum disorder	4 (0.5)	0 (0.0)	NA	0 (0.0)	1 (0.5)	0.313	3 (1.3)	0.089
Sensorineural defects	11 (1.3)	5 (2.3)	**0.025**	0 (0.0)	1 (0.5)	0.313	5 (2.2)	**0.028**
*Morbidity, minor*								
Any minor disorder	36 (4.1)	11 (5.0)	0.336	7 (3.2)	11 (5.1)	0.321	7 (3.1)	0.939
Specific developmental disorders of speech and language	18 (2.1)	8 (3.7)	0.055	2 (0.9)	6 (2.8)	0.148	2 (0.9)	0.968
Specific developmental disorder of motor function	14 (1.6)	2 (0.9)	1.000	2 (0.9)	4 (1.9)	0.401	6 (2.6)	0.171
Mixed specific developmental disorder	8 (0.9)	2 (0.9)	0.653	3 (1.4)	3 (1.4)	0.986	0 (0.0)	0.076
Attention-deficit hyperactivity disorders	2 (0.2)	0 (0.0)	0.317	1 (0.5)	1 (0.5)	0.992	0 (0.0)	0.307
Delayed milestone in childhood	20 (2.3)	4 (1.8)	0.522	6 (2.8)	4 (1.9)	0.537	6 (2.6)	0.943

Bold values indicate statistical significance *p* < 0.05.

*NA* not applicable, *us-EPO* umbilical cord serum erythropoietin.

^a^Test for relative propotions. Reference group is us-EPO 20–39.

Association of us-EPO levels with neurodevelopmental morbidity and infant mortality were analyzed by logistic regression using multivariate enter models adjusted for gestational age with term group as a reference (Table 3). The results are shown as odds ratios (OR) with 95% confidence intervals (CI). A comparison of the distribution of us-EPO levels was made by using Kolmogorov–Smirnov two-samples test (Fig. 2). Correlation analysis was performed by Pearson correlation coefficients.

**Table 3:** Risk for adverse outcomes according to us-EPO categories by logistic regression.

Outcome	Total	us-EPO level at birth											
		Low < 20			Average 20–39			Elevated 40–100			High > 100		
	*n* (%)	*n* (%)	OR^a^	(95% CI)	*n* (%)	OR^a^	(95% CI)	*n* (%)	OR^a^	(95% CI)	*n* (%)	OR^a^	(95% CI)
Mortality													
Died at the age of 0–364 days	12 (1.4)	3 (1.4)	0.87	(0.14–5.56)	2 (0.9)	1.0	(Reference)	3 (1.4)	1.84	(0.30–11.36)	4 (1.8)	1.60	(0.28–9.13)
Morbidity, major													
Any major impairment	26 (3.0)	9 (1.5)	4.32	(0.91–20.44)	2 (2.6)	1.0	(Reference)	5 (3.5)	2.67	(0.51–13.96)	10 (7.4)	**4.87**	**(1.05–22.58)**
Morbidity, minor													
Any minor impairment	36 (4.1)	11 (5.0)	1.17	(0.43–3.18)	7 (3.2)	1.0	(Reference)	11 (5.1)	1.78	(0.67–4.73)	7 (3.1)	0.93	(0.32–2.75)

Statistically significant association is presented in bold.

*CI* confidence interval, *OR* odds ratio, *us-EPO* umbilical cord serum erythropoietin.

^a^Odds ratios (95% confidence interval) are adjusted for gestational age.

Statistical analyses were performed with IBM SPSS Statistics version 20.0 (2014) and SAS version 9.4 (SAS Institute Inc., Cary, North Carolina). *p*-values lower than 0.05 were considered statistically significant.

## Results

### Clinical characteristics

Of a total of 878 live-born children, 11.4% (*n* = 100) were born preterm, 11.6% (*n* = 102) post-term, and 3.8% (*n* = 33) from multiple pregnancies. The mean gestational age was 39.6 (SD 2.8) weeks. The study population included 866 mothers with a mean age of 31.7 (SD 5.3) years. Maternal gestational diabetes incidence was 15.0%, diabetes type I 3.0%, hypertension 3.0%, and pre-eclampsia 4.1%. Birth asphyxia was diagnosed in 21.8% (*n* = 191). Characteristics of newborns are shown in Table 1. Maternal characteristics are presented in the supplement.

### Erythropoietin

The median us-EPO level was 40.8 IU/l (interquartile range 20.3–104.0 IU/l). In the study population, there were 227 (25.9%) children with high us-EPO levels (>100 IU/l). Of these children, 81 (9.2%) had us-EPO levels >500 IU/l and 54 (6.2%) children >1000 IU/l. us-EPO levels <10 IU/l were observed in 66 (7.5%) children.

Children with high us-EPO levels were more likely to be born by cesarean section (64.3% vs. 25.7%, *p* < 0.001), to have a lower mean birth weight (3307 vs. 3504 g, *p* *=* 0.001), and have an Apgar score <7 at 1 (76.2% vs. 52.3%, *p* < 0.001) and 5 min (46.7% vs. 25.7%, *p* *<* 0.001) than children with us-EPO 20–39 IU/l. These children were also more likely to be admitted to the neonatal unit than children with us-EPO 20–39 IU/l (61.7% vs. 29.8%, *p* < 0.001). Children with low us-EPO levels were more likely to be born preterm (19.3% vs. 7.3%, *p* < 0.001), less likely to be born post-term (3.2% vs. 10.6%, *p* *=* 0.002), and more likely had a lower mean birth weight (3224 vs. 3504 g, *p* *<* 0.001) than children with us-EPO 20–39 IU/l (Table 1).

**Fig. 1:**
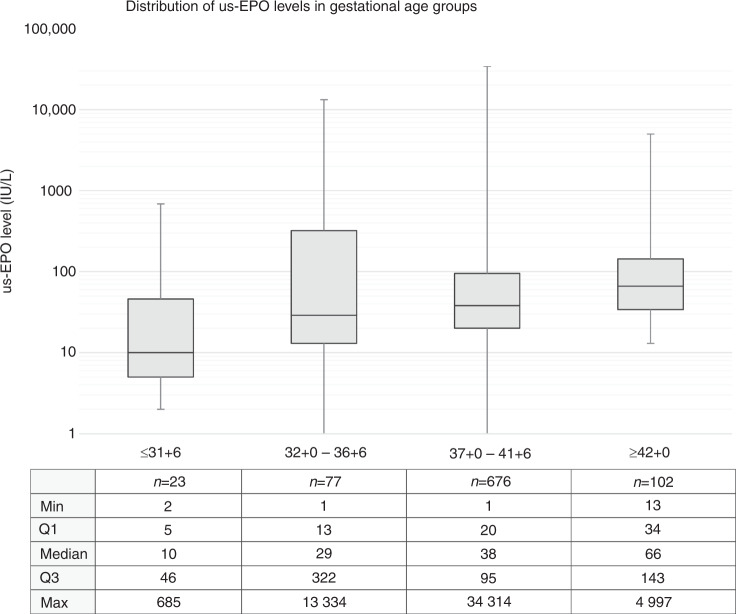
Distribution of umbilical cord serum erythropoietin (us-EPO) levels in different gestational age groups. us-EPO levels are plotted on a logarithmic scale and data on group size and quartiles of us-EPO in each group are shown in the table below the graph.

Us-EPO levels correlated with gestational age (r = 0.526). The median us-EPO level increased by gestational age (Fig. 1).

### Neurodevelopmental morbidity and mortality

At the end of 2018, of a total of 878 children, 18.1% (*n* = 159) were under 3 years of age, 16.5% (*n* = 145) were 3 years of age, 12.2% (*n* = 107) were 4 years of age, 38.2% (*n* = 335) were 5 years of age, and 13.7% (*n* = 120) were 6 years of age. After combining the us-EPO data with registers, 82 (9.3%) children were identified with any neurodevelopmental diagnosis. At least one major neurodevelopmental impairment was identified in 26 (3.0%) children, of whom 1 (3.8%) child was born early preterm, 4 (15.4%) children were born late preterm, 19 (73.1%) children were born term, and 2 (7.7%) post-term. Of these 26 children, 4 children had two major diagnoses and 1 child had four major diagnoses. The most common severe neurodevelopmental impairment in the study population was sensorineural defects (*n* = 11, 1.3%), consisting of 7 (0.8%) children with visual impairment and 4 (0.5%) children with hearing impairment. No child had both visual and hearing impairment. In addition, 36 (4.1%) children had at least one minor neurodevelopmental disorder. In the other 20 (2.3%) children, development was delayed without an as-yet more specific diagnosis. (Table 2)

A total of 12 (1.4%) live-born children died; most of them (75.0%) died during the perinatal period. Three newborn infants died during the first day of life, 6 died at the age of 1–6 days, 1 child died later during the first month, and 2 died later in the first year of life.

### Results from analyses

**Fig. 2:**
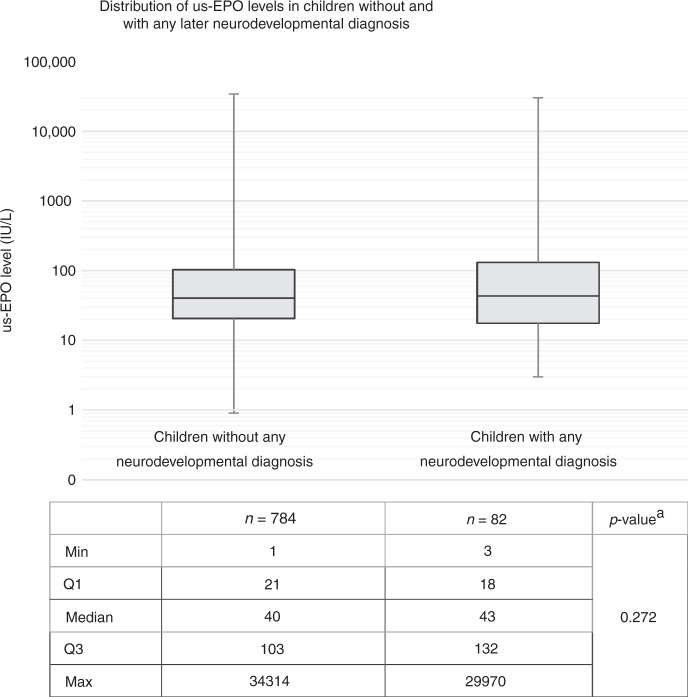
Distribution of umbilical cord serum erythropoietin (us-EPO) levels at birth in children without and with any later neurodevelopmental diagnosis. Neurodevelopmental morbidity includes cerebral palsy, epilepsy, intellectual disability, autism spectrum disorders, sensorineural defects, and minor neurodevelopmental disorders. Infant deaths in the first year (*n* = 12) are excluded. us-EPO levels are plotted on a logarithmic scale and data on group size and quartiles of us-EPO in both groups are shown in the table below the graph. A comparison of the distribution of us-EPO levels between groups was made by using. ^a^Kolmogorov–Smirnov two-sample test.

A severe neurodevelopmental impairment was more likely to be diagnosed in children with an abnormal us-EPO, whether low (4.1% vs. 0.9%, *p* *=* 0.033) or high (4.4% vs. 0.9%, *p* *=* 0.023), than in children with us-EPO 20–39 IU/l (Table 2). High us-EPO levels (>100 IU/l) at birth were associated with an increased risk of severe neurodevelopmental morbidity (OR 4.87, 95% CI 1.05–22.58) in the model adjusted for the gestational age. No association of high us-EPO with minor neurodevelopmental disorders or with infant mortality was observed (Table 3). No statistically significant difference in the distribution of us-EPO levels was found between children with or without any neurodevelopmental diagnosis (Fig. 2).

## Discussion

In this study, we determined whether endogenous us-EPO is a useful biomarker to assess and predict the risk of neurodevelopmental morbidity. Our hypothesis was that not only severe neurodevelopmental diseases but also minor neurodevelopmental disorders and mortality occur more often in children with high us-EPO than children with average us-EPO.

We observed that high us-EPO concentration at birth, regardless of gestational age, was associated with an increased risk of major neurodevelopmental morbidity in early childhood. No statistically significant association occurred between us-EPO and minor neurodevelopmental disorders. High us-EPO was not associated with infant mortality. We observed that the us-EPO level at birth was dependent on gestational age; the lower the gestational age, the lower the median us-EPO level.

### Comparison with previous findings

To our knowledge, only two studies have previously assessed the significance of high endogenous EPO levels of newborns in subsequent neurodevelopmental morbidity. One follow-up study from 1988 revealed that high umbilical plasma EPO levels after acute birth asphyxia (*n* = 31, of whom 5 preterm children), but not after pre-eclampsia (*n* = 62, of whom 42 preterm children), were associated with an increased risk of cerebral palsy, a Bayley Mental Developmental Index below 90, or death at the 2 years of age.^13^ Our results are rather similar to these preliminary results obtained with a small study population, although our analyzes were not limited to the asphyxia group alone. Korzeniewski et al. determined endogenous EPO levels on day 14 from 786 infants born before the 28th week of gestation. The highest quarter of endogenous EPO levels were associated with very low (< 55) Mental (OR 2.3; 95% CI 1.5–3.5) or Psychomotor (OR 2.4; 95% CI 1.6–3.7) Development Indices (or both) at 2 years of age.^14^ In our study, blood samples were collected immediately after birth in order to more accurately assess the significance of us-EPO concentration in relation to oxygen deficiency at birth. Our cohort also consisted mostly of children (88.6%) born term or post-term. In contrast to these two previous studies, we assessed the association of us-EPO with precisely defined diagnoses, and at the time of evaluation, most children (51.9%) were over 5 years of age.

In addition, Forestier et al. reported that fetal EPO levels remained stable throughout pregnancy with no correlation between EPO and gestational age, when EPO levels were measured in 163 fetal intrauterine blood samples from the 18th week of gestation to the end of pregnancy.^21^ Subsequently, Seikku et al. found us-EPO to correlate with gestational age among vaginal term and post-term births (*r* = 0.250),^24^ while Bahr et al. reported no difference in us-EPO levels between term (*n* = 13) and preterm (*n* = 10).^25^ Our results confirmed gestational age-dependent correlation in EPO levels, when EPO was determined from umbilical cord serum samples at birth.

### Deliberation

Hypoxia causes brain damage. Since EPO levels increase due to hypoxia, EPO could be a useful biomarker to predict later neurodevelopmental problems. On the other hand, several in vitro and in vivo studies have shown the effects of EPO to be anti-apoptotic, anti-inflammatory, and antioxidative.^4,26–28^ EPO also promotes brain revascularization and neurogenesis.^4,5,26–28^ Preliminary results in children suggest that EPO is also involved in the regulation of systemic inflammation and may thereby contribute to subsequent events. Levels of EPO in blood on postnatal days 1, 7, and 14 vary monotonically with inflammation-related proteins in extremely preterm children.^29^ High EPO combined with high cytokine concentrations in the blood sample taken during the first postnatal month has been associated with an increased risk of ADHD symptoms in 10-year-old children born extremely preterm.^30^ Zareen et al. assessed school-age children with a background of neonatal encephalopathy (NE) and age-matched controls for their cytokine responses and neurodevelopmental outcome.^31^ They demonstrated that EPO levels in post-NE children were higher compared with controls, especially in response to in vitro stimulation with lipopolysaccharide.

The evidence of a neuroprotective role of EPO is controversial: recent randomized, placebo-controlled trials have shown no benefit of high-dose EPO treatment in improving neurological outcome.^8–10^ Our finding of the association of high us-EPO levels with severe neurodevelopmental morbidity may indicate that, despite the neurotrophic effects of EPO, a significant portion of children still develop brain damage when hypoxia has been intense enough to increase us-EPO to a very high level. In our study, a fairly large number of asphyxia diagnoses (22%) probably reflects the inclusion criteria of low Apgar scores in selecting the cohort.

Another consideration is timing. It has previously been reported that plasma EPO levels do not start to increase until ~90 min after the beginning of acute hypoxia in human adults^32^ and within 2–3 h in fetuses in animal experiments.^33,34^ The peak values of EPO levels are reached in 1–2 days and fall thereafter.^1,35^ EPO is not stored in tissues and the half-life of EPO in the blood is 2–4 h in newborn infants.^36^ Thus, even if the newborn infant suffers from the lack of oxygen during childbirth, if the delivery is rapid, us-EPO levels may not yet show the entire magnitude of increase at the time of birth. In addition, umbilical cord blood has been shown to differ significantly from the subsequent postnatal blood sample, inter alia, in cell composition, plasma protein concentrations, and immune cell phenotypes.^37^ A second postnatal sample could thus be beneficial and substantive in the timing of hypoxia and in evaluating the significance of EPO as a marker of birth asphyxia.

When interpreting the us-EPO level in clinical use, gestational age should be considered, because it appears that birth itself, or associated with hypoxia, may not increase the us-EPO level in premature children. Overall, the role of endogenous us-EPO to predict the risk of neurodevelopmental morbidity in clinical use is likely to be minor and without careful evaluation may even lead to misinterpretation and misdirect the child’s follow up.

### Limitations and strengths

Due to the standard practice of collecting umbilical blood samples immediately after birth, we used 1-min Apgar scores to identify newborns to enroll in the study, although 5-min scores are known to better predict long-term outcomes.^38,39^ The notable limitation of our study was that 27% of the 5-min Apgar scores were unrecorded due to the common practice of not providing a 5-min score for children with a high 1-min score. Based on clinical experience, there was no medical need to record 5-min scores in these cases.

In addition, we had no data on obstetric and neonatal complications, such as previous transient fetal bleeding or erythema infectiosum infection, or durations of deliveries that could affect us-EPO levels. Data on neonatal interventions, which may reduce the risk of death and adverse neurodevelopmental outcome, such as induced hypothermia for children with hypoxic–ischemic encephalopathy, were also not available. It should also be noted that only severe impairments can be diagnosed during the first few years of life. Hyperkinetic disorders, specific developmental disorders, and mild intellectual disability can often only be detected later. Nevertheless, the strength of this study was a study population of good size that was not limited to risk groups. Thus, the results are better generalizable to the population level. The data were collected from high-quality and comprehensive registers^15–17^ and diagnoses were specifically defined and always made by a specialist in neurodevelopmental disorders. The study design allowed for the clinical relevance of endogenous us-EPO to be assessed.

## Conclusion

us-EPO levels were dependent on gestational age and were lower in premature children, which should be considered when interpreting the us-EPO level. Although high us-EPO concentration at birth was associated with an increased risk of neurodevelopmental morbidity in early childhood regardless of gestational age, determining endogenous us-EPO appears to be of minor clinical utility.

## Supplementary information

Supplemental_Table

Figure t

Figure
