# Supplementary material for: Endogenous erythropoietin at birth is associated with neurodevelopmental morbidity in early childhood
Source: Pediatr Res. 2021 Aug 31;92(1):307–14. doi: 10.1038/s41390-021-01679-0 (PMC9411059; doi:10.1038/s41390-021-01679-0)
Supplement: Supplementary file 1 — Supplemental_Table [file 41390_2021_1679_MOESM1_ESM.docx]

| **Supplemental Table. Maternal characteristics of the study population, overall and stratiﬁed by us-EPO categories.** | | | | | | |  |  |
| --- | --- | --- | --- | --- | --- | --- | --- | --- |
|  | **Total** | **us-EPO level at birth** | | | | | | |
|  |  | **Low <20** | | **Average 20-39** | **Elevated 40-100** | | **High >100** | |
| **Characteristics** | **n (%)** | **n (%)** | ***p*-value^a^** | **n (%) Reference group** | **n (%)** | ***p*-value^a^** | **n (%)** | ***p*-value^a^** |
| Total number of children | 878 (100.0) | 218 (100.0) |  | 218 (100.0) | 215 (100.0) |  | 227 (100.0) |  |
| Maternal socioeconomic position^b^ |  |  |  |  |  |  |  |  |
| Blue collar | 121 (13.8) | 28 (12.8) | 0.247 | 33 (15.1) | 29 (13.5) | 0.799 | 31 (13.7) | 0.987 |
| Lower white collar | 125 (14.2) | 29 (13.3) |  | 33 (15.1) | 31 (14.4) |  | 32 (14.1) |  |
| Higher white collar | 51 (5.8) | 9 (4.1) |  | 17 (7.8) | 13 (6.0) |  | 12 (5.3) |  |
| Other | 581 (66.2) | 152 (69.7) |  | 135 (61.9) | 142 (66.0) |  | 152 (67.0) |  |
| Maternal BMI (kg/m²) |  |  |  |  |  |  |  |  |
| Overweight: BMI 25.0-29.9. | 192 (21.9) | 42 (19.3) | 0.409 | 49 (22.5) | 42 (19.5) | 0.452 | 59 (26.0) | 0.387 |
| Obese: BMI ≥ 30.0. | 101 (11.5) | 25 (11.5) | 0.261 | 18 (8.3) | 29 (13.5) | 0.080 | 29 (12.8) | 0.121 |
| Maternal smoking |  |  |  |  |  |  |  |  |
| No smoking during pregnancy | 775 (88.3) | 194 (89.0) | 0.240 | 192 (88.1) | 188 (87.4) | 0.654 | 201 (88.5) | 0.727 |
| Quit smoking at 1st trimester | 37 (4.2) | 10 (4.6) |  | 7 (3.2) | 11 (5.1) |  | 9 (4.0) |  |
| Current smokers | 46 (5.2) | 8 (3.7) |  | 16 (7.3) | 12 (5.6) |  | 10 (4.4) |  |
| No information | 20 (2.3) | 6 (2.8) |  | 3 (1.4) | 4 (1.9) |  | 7 (3.1) |  |
| Maternal diseases |  |  |  |  |  |  |  |  |
| Gestational diabetes (O24.4, O24.9) | 132 (15.0) | 28 (12.8) | 0.411 | 34 (15.6) | 37 (17.2) | 0.650 | 33 (14.5) | 0.755 |
| Diabetes type I (E10, O24.0) | 26 (3.0) | 6 (2.8) | 0.778 | 7 (3.2) | 4 (1.9) | 0.372 | 9 (4.0) | 0.669 |
| Hypertension (O13) | 26 (3.0) | 1 (0.5) | **0.018** | 8 (3.7) | 11 (5.1) | 0.462 | 6 (2.6) | 0.535 |
| Pre-eclampsia (O14) | 36 (4.1) | 6 (2.8) | 0.308 | 10 (4.6) | 9 (4.2) | 0.839 | 11 (4.8) | 0.898 |
| In vitro fertilization | 36 (4.1) | 8 (3.7) | 0.805 | 9 (4.1) | 7 (3.3) | 0.630 | 12 (5.3) | 0.565 |
| Parity |  |  |  |  |  |  |  |  |
| 0 | 507 (57.7) | 113 (51.8) | 0.066 | 110 (50.5) | 137 (63.7) | **0.017** | 147 (64.8) | 0.880 |
| 1 | 252 (28.7) | 61 (28.0) |  | 79 (36.2) | 54 (25.1) |  | 58 (25.6) |  |
| 2 or more | 119 (13.6) | 44 (20.2) |  | 29 (13.3) | 24 (11.2) |  | 22 (9.7) |  |
| Induced delivery | 308 (35.1) | 63 (28.9) | 0.301 | 73 (33.5) | 83 (38.6) | 0.267 | 89 (39.2) | 0.210 |
| Data are presented as variables and percentage. | | | | | | | | |
| *BMI* body mass index, *us-EPO* umbilical cord serum erythropoietin. | | | | | | | | |
| ^a^Test for relative propotions, Chi-square test or t-test. Reference group is us-EPO 20-39. | | | | | | | | |
| ^b^Socioeconomic position was classified into four categories based on the mother’s occupation: upper white-collar workers, such as physicians and teachers; lower white-collar workers, such as nurses and secretaries; blue-collar workers, such as cooks and cleaners; and others including for example entrepreneurs and students. (Gissler M. et al. Trends in socioeconomic differences in Finnish perinatal health 1991-2006. *J Epidemiol Community Health* 63, 420–5 (2009)) | | | | | | | | |
| Bold values indicate statistical significance *p* < 0.05. | | | | | | | | |
