# Supplementary figures and images for: Endogenous erythropoietin at birth is associated with neurodevelopmental morbidity in early childhood
Source: Pediatr Res. 2021 Aug 31;92(1):307–14. doi: 10.1038/s41390-021-01679-0 (PMC9411059; doi:10.1038/s41390-021-01679-0)

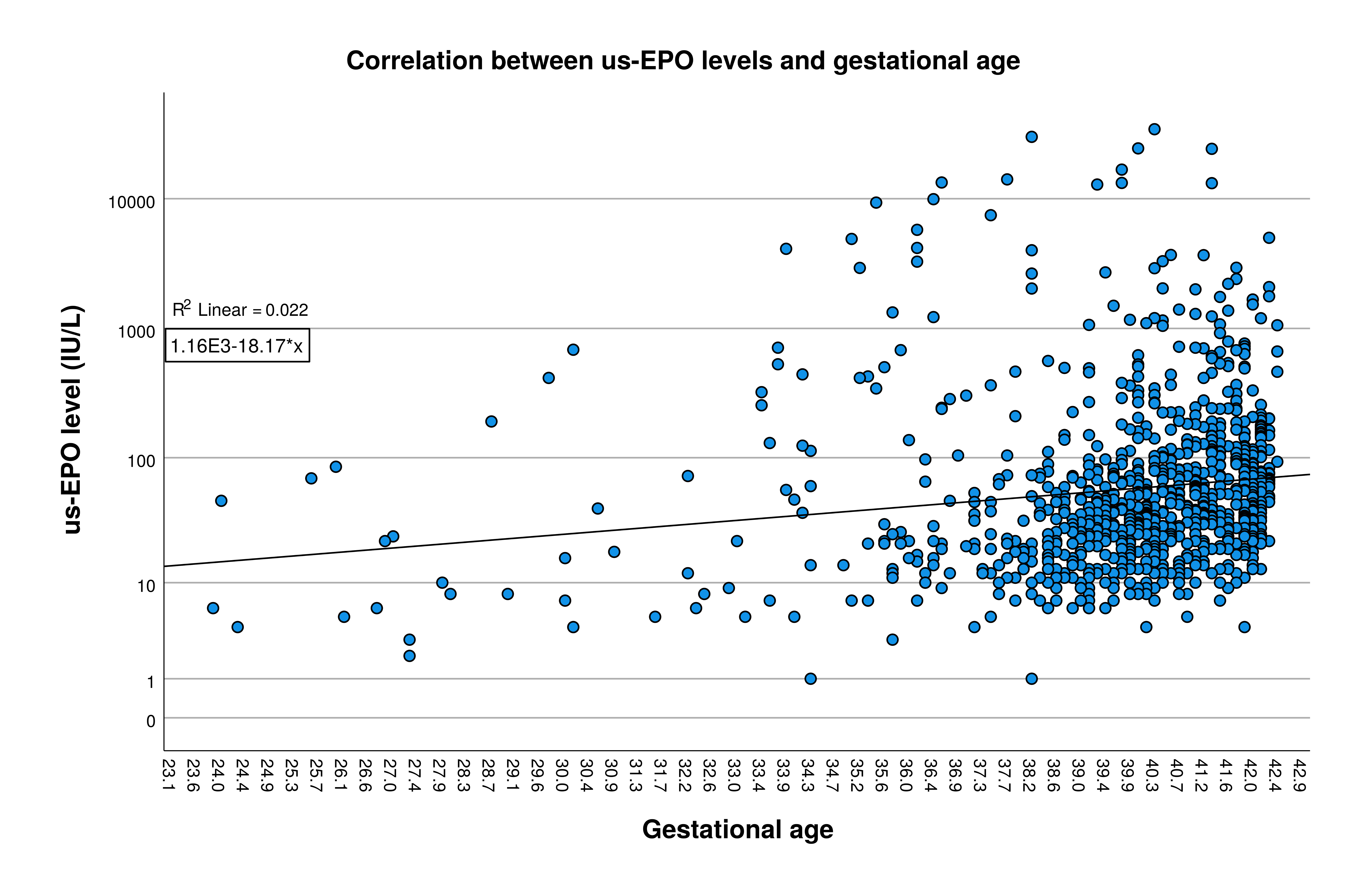

Supplement: Supplementary file 2 — Figure t [file 41390_2021_1679_MOESM2_ESM.tif]

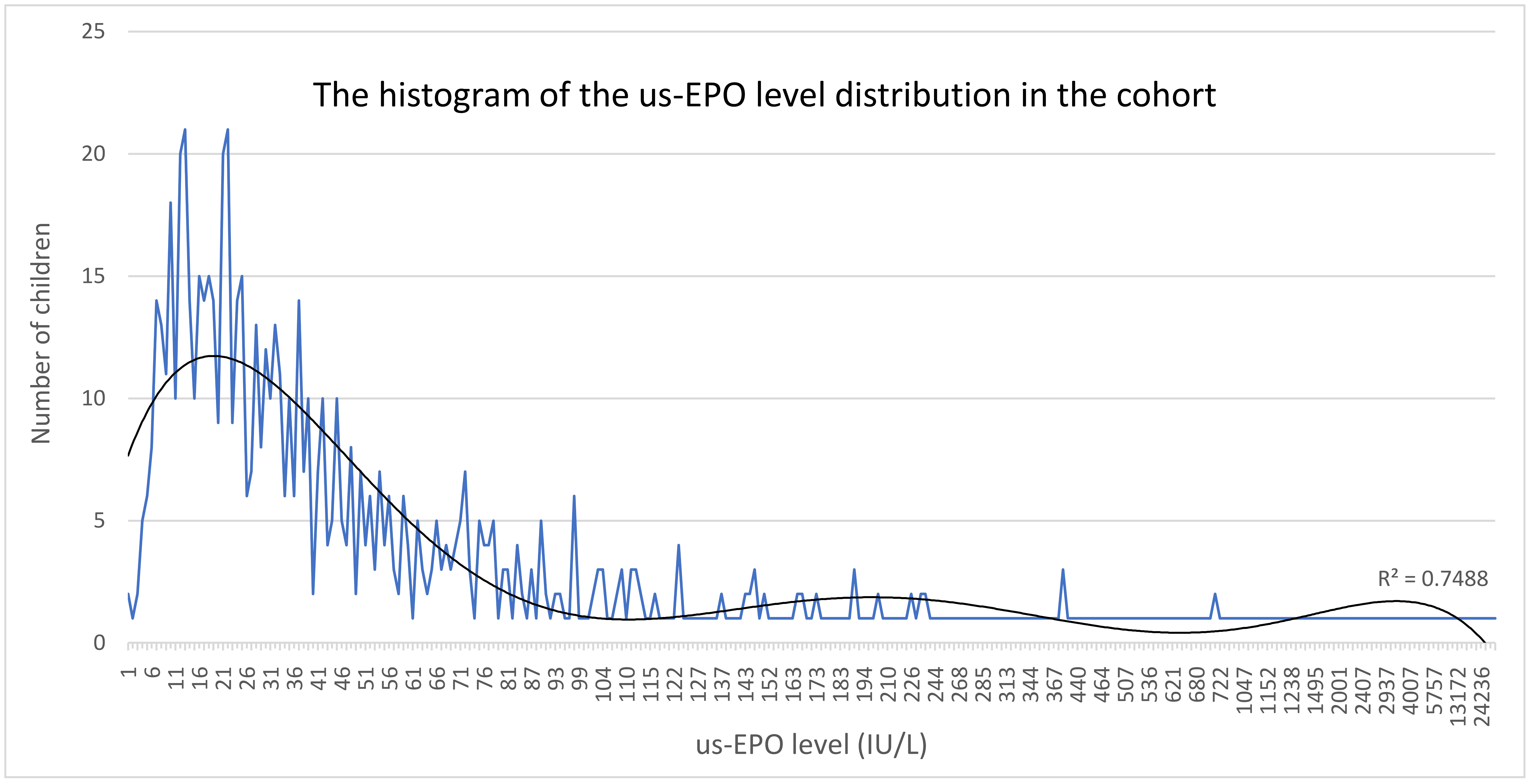

Supplement: Supplementary file 3 — Figure [file 41390_2021_1679_MOESM3_ESM.tif]
